# Effects of acute single-session exercise modalities on stroop test performance in male adolescents: an exploratory study using the nonparametric cognitive diagnosis model

**DOI:** 10.3389/fspor.2026.1883836

**Published:** 2026-10-27

**Authors:** Jiun-Jie Wang, Cheng-Hsuan Li, Wen-Hsin Chiu, Chen-Sin Hung

**Affiliations:** 1 Graduate Institute of Educational Information and Measurement, National Taichung University of Education, Taichung, Taiwan; 2 Department of Kinesiology, National Tsing Hua University, Hsinchu, Taiwan; 3 Department of Physical Education and Sports Sciences, National Taiwan Normal University, Taipei, Taiwan

**Keywords:** aerobic exercise, agility exercise, non-parametric cognitive diagnosis model, Q-matrix, resistance training, stroop test

## Abstract

**Introduction:**

This study investigated the effects of different acute exercise modalities on attention–related cognitive performance and explored the applicability of the Non–parametric Cognitive Diagnosis Model (NPCD) for classifying relative cognitive attribute mastery patterns.

**Methods:**

Sixty healthy 18-year-old male high school students were assigned to one of four groups: Agility Exercise (GE), Resistance Exercise (RE), Aerobic Exercise (AE), and No Exercise Control (NE), with 15 participants in each group. Participants completed the Stroop Color-Word Test (SCWT) under static, mobile, and complex attention conditions before and after the intervention. Post-test performance was analyzed using analysis of covariance (ANCOVA), with baseline scores entered as covariates. NPCD analysis, combined with a Q-matrix framework, was used to classify relative cognitive mastery patterns in an exploratory manner.

**Results:**

After controlling for baseline performance, significant group effects were observed for static attention, *F*(3,55) = 84.389, *p* < .001, *η*² = .822; mobile attention, *F*(3,55) = 28.466, *p* < .001, *η*² = .608; and complex attention, *F*(3,55) = 44.886, *p* < .001, *η*² = .710. Large effect sizes were observed across all attention conditions (*η*² = .608-.822). GE demonstrated superior performance in static and mobile attention, whereas AE showed the greatest improvement in complex attention. NPCD analysis further identified distinct relative cognitive mastery patterns across participants, providing exploratory information regarding individual cognitive attributes.

**Discussion:**

The findings indicate that acute exercise modalities may exert distinct effects on attentional performance, with agility exercise showing greater benefits for static and mobile attention and aerobic exercise for complex attention. The integration of NPCD with a Q-matrix may provide a supplementary exploratory approach for examining individual cognitive attribute patterns beyond group-level performance. Given the homogeneous male adolescent sample and exploratory nature of the NPCD classifications, these findings should be validated in larger and more diverse populations function.

## Introduction

Recent research has established physical exercise as a key determinant of both physiological and cognitive health. Emerging evidence suggests that distinct exercise modalities differentially influence attention and executive functions–core components of higher-order cognition (1, 2). Aerobic, resistance, and agility training have been extensively examined for their effects on cognitive functions, particularly attention and executive function. Notably, agility training has attracted growing interest for its multidimensional benefits to both physical and cognitive performance (2, 3). Reactive agility is the capacity to rapidly modify movements in response to unpredictable stimuli or environmental changes, particularly in dynamic sports contexts (4). Agility training involves the rapid and efficient alteration of direction, speed, and posture, integrating speed, strength, coordination, balance, and reaction time. It aims to enhance responsiveness to external stimuli, minimize injury risk, and optimize performance in sports requiring quick directional changes (5). From neurophysiological perspective, agility is an integrative ability combining neuromuscular and cognitive processes, enabling rapid information processing and precise motor responses (6). Fundamental principles of agility training emphasize rapid changes of direction, high-intensity short-duration efforts, and repetition. Effective training incorporates brief, maximal multi-directional movements (within∼20 s) to enhance sport-specific agility while preserving neuromuscular control (2). Repetition strengthens neural pathways between the central and muscular systems, thereby improving motor coordination and reducing reaction time (7). Effective agility performance relies on reaction speed, movement velocity, explosiveness, coordination, and balance. Agility training enhances these abilities by demanding rapid responses to unpredictable stimuli, strengthening both cognitive and muscular responsiveness (4, 5).

In summary, agility represents an integrated interaction of physical and cognitive systems. Reactive agility training enhances attentional control, movement precision, and sport-specific performance by facilitating faster neural processing and motor execution (2, 4). Reactive agility performance more effectively distinguishes athletes of different skill levels than change-of-direction tests (8). This study investigates the effects of aerobic exercise, resistance training, and agility training on attentional cognitive functions using the Stroop Color-Word Test (SCWT). It was used to assess executive function, selective attention, and inhibitory control. Participants were required to identify the color of words while inhibiting the automatic tendency to read the word itself. Performance was evaluated based on response time, with greater interference effects indicating poorer inhibitory control. The SCWT is a well-established and widely validated neuropsychological measure of cognitive control (9). By comparing pre- and post-intervention outcomes, it aims to identify the distinct contributions of each modality to attention and executive function. Aerobic exercise, characterized by elevated heart rate and respiration, enhances cardiopulmonary efficiency through improved oxygen transport and energy metabolism, contributing to greater endurance and reduced cardiovascular risk (10). Meta-analyses suggest that aerobic training enhances cognition by increasing brain-derived neurotrophic factor (BDNF), promoting neurogenesis, and improving vascular function. Resistance training, primarily anaerobic, recruits extensive muscle fibers to enhance strength and induce hormonal responses, facilitating hypertrophy, tissue repair, and long-term gains in bone density and metabolic rate (11). Resistance training enhances executive control, decision-making, and emotion regulation, while fostering greater self-efficacy and confidence (1). Agility training, characterized by rapid neuromuscular activation, improves coordination, balance, and reaction speed through short, high-intensity anaerobic efforts, engaging vestibular and proprioceptive systems to enhance acceleration, deceleration, and directional control (12). Agility training significantly enhances reaction speed, selective attention, and decision-making, with dual-task approaches shown to improve both physical and cognitive performance in older adults (13). Reactive agility with cognitive elements shows promise for fall prevention in older adults (14). Six-week interventions have been shown to enhance selective attention and emotional regulation (15). Reactive agility performance primarily relies on cognitive factors, including reaction time and decision-making, highlighting the interdependence of physical and cognitive demands (16, 17). Both physical and cognitive factors must be considered in designing active breaks and assessing performance outcomes. This study incorporated cognitive tasks within three attention paradigms to examine their effects on motor-cognitive reactive agility. Comparative analyses revealed that agility training, based on Illinois Agility Run Test (IART) standards, produced the most significant cognitive improvements (*p* < .05) (18). The IART is a standardized field assessment evaluating speed, body control, and directional change efficiency. Conducted on a 10 × 5 m course marked with cones, participants start prone, sprint, return, zigzag through central cones, and sprint to finish. Completion time, measured to the nearest 0.1 s, indicates agility performance. Normative values show male athletes typically finish in 15.2–17.0 s, and females in 17.0–19.0 s (19). The IART shows high test–retest reliability (ICC > 0.90) across diverse populations (20). The IART is a validated and standardized measure of agility in sports science and physical education. Participants completed the IART twice during before and after the agility training session intervention. Participants not meeting relative mastery criteria received remedial instruction via a non-parametric cognitive diagnosis model, after which performance improved. The study aimed to clarify how each exercise modality enhances attention and executive functions. Based on previous findings regarding the cognitive benefits of aerobic, resistance, and agility exercise, based on the existing literature regarding exercise-induced cognitive enhancement and cognitive diagnostic assessment, two research hypotheses were proposed.

H1: Different exercise modalities will produce differential effects on attention performance as measured by the Stroop Color–Word Test.
H2: The Non-Parametric Cognitive Diagnosis Model (NPCD) will identify distinct relative mastery and relative non-mastery patterns across multiple cognitive attributes.

## Materials and methods

### Participants recruitment

Sixty healthy 18-year-old male students from Ruifang Senior Vocational Industrial High School voluntarily participated. None had exercised regularly in the past three months (non-periodic movement pattern). Inclusion criteria excluded individuals with chronic diseases, visual impairments, or neurological or psychiatric disorders, and all answered “no” to the PAR-Q items. The study was approved by the Institutional Review Board of Chung Cheng University, Chiayi, Taiwan (IRB−2026-114110601) and conducted in accordance with the Declaration of Helsinki. An *a priori* power analysis was conducted using G*Power 3.1 to estimate the required sample size for comparisons among four independent groups. Assuming *α* = .05, statistical power = .80, and a medium-to-large expected effect size based on previous studies examining acute exercise effects on cognitive performance, the required total sample size was estimated to be approximately 56–60 participants. Therefore, the recruitment of 60 participants, with 15 participants assigned to each group, was considered sufficient for the planned between-group comparisons.

### Experimental procedure

This study was conducted at Ruifang Senior Vocational Industrial High School between March to May 2026.

The experiment comprised four groups-Agility (GE), Resistance (RE), Aerobic (AE), and No exercise (NE)-with 15 participants per group. The SCWT was administered before and after each intervention under static, mobile, and complex attention conditions to assess exercise-induced cognitive changes. Time (pre-test vs. post-test) was treated as a within-subjects factor, participants were assigned to only one exercise condition, and the SCWT performance was assessed before and after the assigned intervention, and each participant completed only one intervention condition throughout the study. No participant crossed over to another intervention condition, and each participant completed only the assigned exercise protocol. Participants avoided strenuous activity, alcohol, caffeine, and medications 24 h before testing. Experiments were held in a quiet, temperature-controlled (24–26 °C) environment between 8 a.m. and 12 p.m. During the first visit, participants received procedural explanations and underwent baseline assessments, including VO₂ peak, 12RM (upper body), and body composition via bioelectrical impedance. They also practiced the SCWT until achieving ≥85% accuracy. Completion time on the SCWT inherently reflects accuracy, as participants could only proceed after answering each pattern item correctly, with performance evaluated primarily based on response time. The exercise intensity was monitored using the Borg Rating of Perceived Exertion (RPE, 6–20 scale) (21, 22). The RPE scale assessed participants’ subjective psychophysiological responses. The experiment began with SCWT instruction, followed by pre- and post-tests surrounding the exercise intervention. After the post-test, the Q-matrix can be used to rapidly identify individuals who scored below the mean, thereby enabling the provision of targeted remedial instruction. The experimental procedure is illustrated in Figure 1.

**Figure 1:**
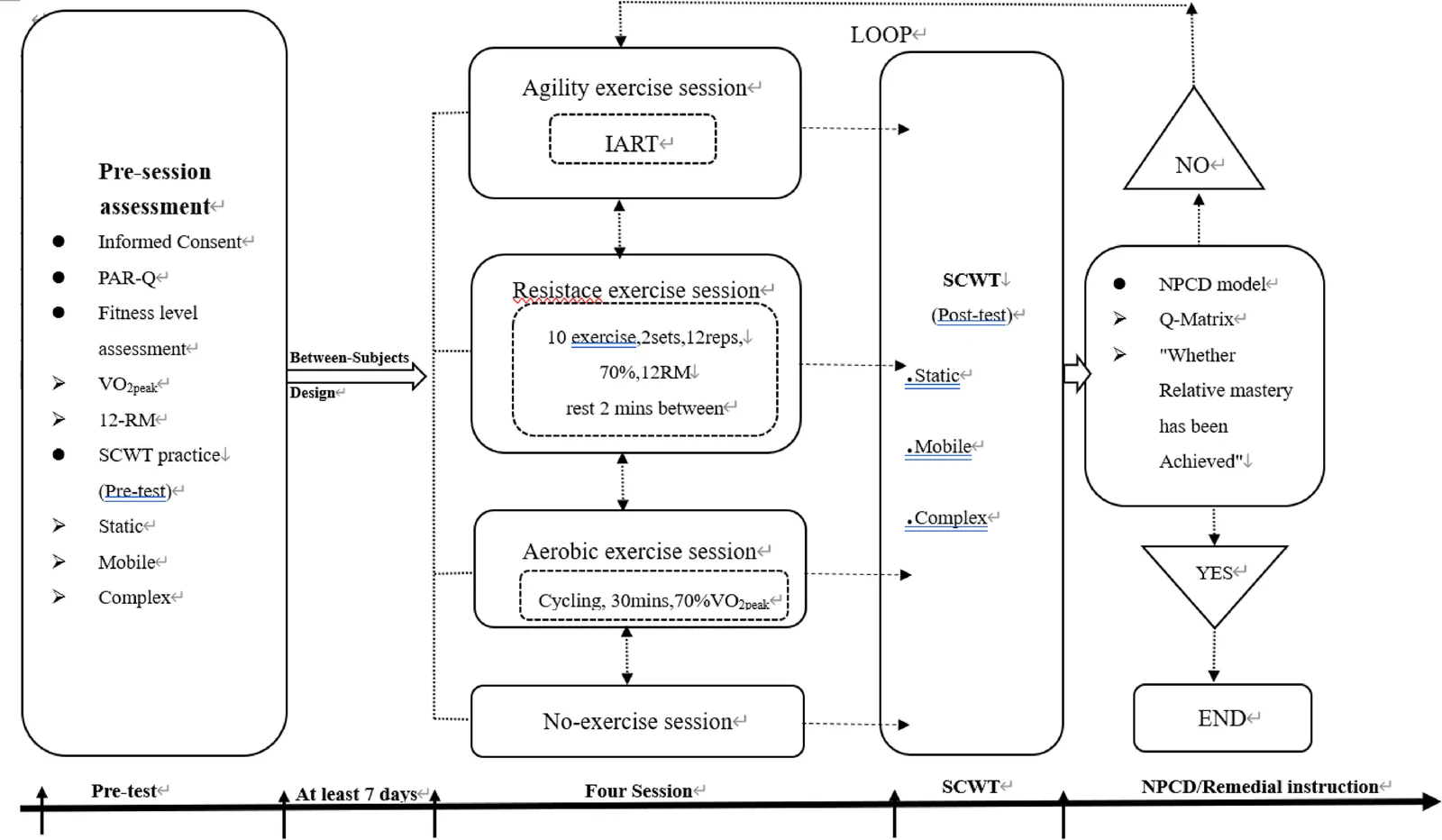
Experimental procedure.

### Sessions

Upon arrival, participants practiced the cognitive task, then wore a heart rate strap. After the first 10 min, they rested for two minutes and consumed 100 mL of water. All sessions were supervised by sports protective officer to ensure proper intensity control and safety.

### Agility exercise session

Agility training entails rapid, whole-body movements involving directional or speed changes in response to external stimuli, integrating physical (balance, coordination, speed) and cognitive (perception, decision-making) components (23). Recent studies show that long-term, multi-component agility training enhances neuromuscular, cardiorespiratory, and cognitive functions, particularly in older adults (12). Agility in this study was assessed using the IART (18). The IART is a reliable, field-based measure of acceleration, includes deceleration, and rapid directional change while maintaining body control. Conducted on a 10 × 5 m flat course marked with cones, participants start prone, sprint, weave through central cones, make 180° turns, and sprint to the finish, decelerations, directional changes, and cone-weaving movements. To ensure that participants were familiar with the entire testing procedure. Instead, participants repeatedly performed IART trials over approximately 30 min. Each maximal trial was followed by a standardized 1-minute passive recovery before the next repetition. This work-rest cycle continued throughout the intervention. The best performance across all trials was selected for subsequent analysis. Due to the IART is a maximal sprint-based agility task, brief recovery intervals are necessary to maintain exercise quality and participant safety. Cognitive assessments were administered immediately before and after the exercise session to examine the acute effects of agility exercise on attention performance. Exercise intensity was maintained at a Borg Rating of Perceived Exertion (RPE) of 10–13, corresponding to moderate intensity. Heart rate was continuously monitored using a chest-strap monitor to ensure consistent exercise intensity across participants. Completion time (in seconds) reflects agility, with shorter times indicating superior performance. The IART used for pre- and post-test assessment was separate from the agility training intervention itself. Owing to its practicality and validity, the IART is widely used in sports science and physical education to assess multidirectional movement and monitor athletic progress.

As shown in Figure 2, participants completed the red course as quickly as possible. For ages 16–19, normative completion times range from 16.2 to 18.1 s (24), as shown in Table 1.

**Table 1:** The evaluation criteria of the IART.

Gender	Excellent	Above Average	Average	Below Average	Poor
Male	<15.2 s	15.2–16.1 s	16.2–18.1sec	18.2–19.3 s	>19.3 s
Female	<17.0 s	17.0–17.9 s	18.0–21.7sec	21.8–23.0 s	>23.0 s

**Figure 2:**
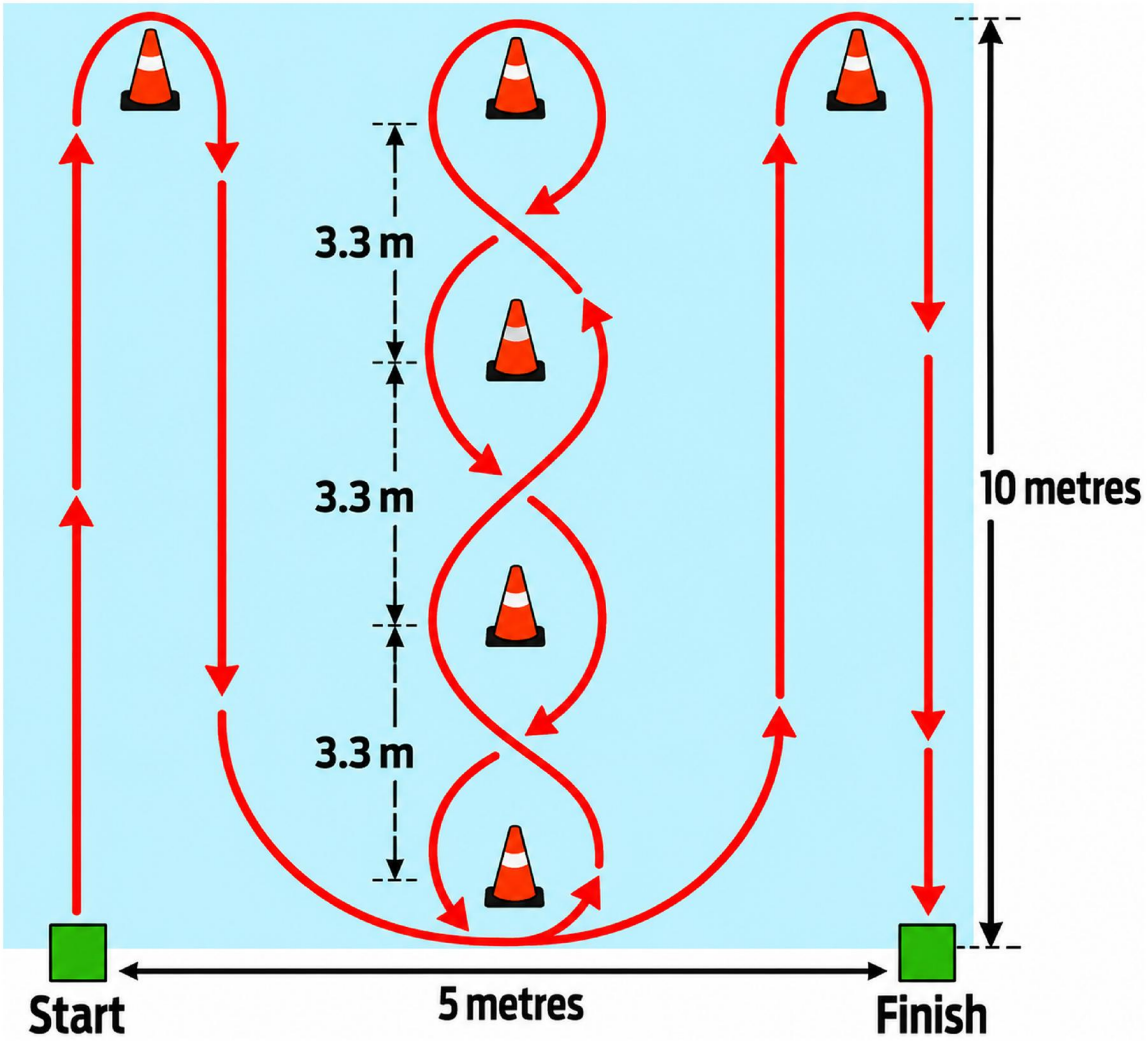
Schematic layout of the IART course.

### Resistance exercise session

In the RE session, participants completed ten resistance exercises targeting major muscle groups, including squat, lunge, leg press, bench press, seated row, shoulder press, biceps curl, triceps extension, abdominal crunch, and back extension. Each exercise was performed for 2 sets of 12 repetitions at approximately 60%–70% of the individually determined 12RM. Two-minute rest intervals were provided between sets and exercises. Total exercise duration was approximately 30 min. Exercise intensity was maintained at an RPE of 10–13 and supervised by sports protective officer to ensure safety and protocol adherence.

### Aerobic exercise session

The AE session involved 30 min of stationary cycling at 60%–70% VO₂ peak. Aerobic exercise primarily utilizes the aerobic energy system, engaging large muscle groups in sustained, rhythmic movements such as cycling or running. It enhances cardiovascular and pulmonary efficiency, improves metabolism, and supports both physical and cognitive performance (25). Thus, 30-minute cycling protocol was employed, demonstrating consistent reliability.

### Stroop color-word test

This study administered the Stroop Color–Word Test (SCWT) using a mobile application. Response time was recorded with a calibrated stopwatch (SEIKO S-141) and verified by the same assistant for inter-rater reliability. The SCWT is a widely used measure of selective (color) attention, inhibitory control, and processing function. Participants were required to identify the font color of each stimulus as quickly and accurately as possible by pressing the corresponding response button on the mobile device. Response time was recorded automatically by the application, and accuracy was calculated as the percentage of correct responses. The use of a smartphone-based Stroop format was informed by previous validation studies demonstrating acceptable validity and reliability in adult clinical populations (26, 27). However, validation evidence specifically involving adolescent athletic populations remains limited, as acknowledged in the Limitations section. Unlike traditional paper-based assessments, this study used a mobile application. A preliminary validation analysis conducted in the present sample showed a strong positive correlation between the paper-based and smartphone-based SCWT formats (r = .856, *p* < .001), supporting measurement consistency between the two versions. Participants identified the font color (e.g., the character “red” shown in blue or green) by pressing the correct button as quickly as possible. Response time reflected task completion speed, whereas accuracy was calculated as the percentage of correct responses. Because participants could proceed to the next item only after providing a correct response, task accuracy was embedded in the completion-time measure. Therefore, completion time was used as the primary performance indicator, with shorter completion times reflecting more efficient and accurate task performance. Therefore, accuracy was not treated as an independent outcome variable.

### Non-parametric cognitive diagnosis model (NPCD) & Q-matrix

The Non-parametric Cognitive Diagnosis Model (NPCD) identifies latent knowledge states without assuming specific statistical distributions. NPCD adopts flexible data-driven methods to classify attribute mastery, making it suitable for educational and psychological assessments (28, 29). The present classification should therefore be regarded as an exploratory, relative, and hypothesis-generating framework rather than a criterion-referenced diagnostic classification. The Q-matrix is a binary matrix defining the relationship between items and required attributes. Each row represents an item and each column an attribute, where “1” denotes relative mastery and “0” non-relative mastery. Accurate cognitive diagnosis relies on the Q-matrix's validity, making its specification and verification essential (30, 31). In the NPCD model (32), Three cognitive attributes were specified in the Q-matrix: (A1) Selective(color) Identification, referring to the ability to accurately identify stimulus colors; (A2) Inhibitory Control, referring to the suppression of automatic word-reading responses during incongruent Stroop trials; and (A3) Processing Speed, referring to the efficiency of responding to visual stimuli under time constraints. Congruent items primarily measured A1, incongruent items measured both A1 and A2, and neutral items primarily reflected A3. The Q-matrix was reviewed by two experts in cognitive assessment and one expert in exercise psychology to establish content validity before analysis. Each Stroop item was mapped onto one or more attributes according to task demands, ensuring conceptual and content validity of the Q-matrix prior to NPCD analysis.

In SCWT-based exercise cognition studies, the NPCD classifies cognitive profiles across dimensions such as selective attention, inhibition, and processing speed. Its non-assumptive structure enhances robustness to variability from exertion, fatigue, and individual differences (33). The determination of relative mastery versus non- relative mastery in NPCD using the Q-matrix was based on consistency, inconsistency, and neutral conditions, while the assessment was conducted across static, dynamic, and complex testing conditions. This approach allows for precise inference of knowledge states by aligning observed responses under multiple test conditions with the item–attribute mappings defined in the Q-matrix (34). After the post-test, the Q-matrix was used to exploratorily classify participants according to relative mastery patterns, providing preliminary information that may inform future individualized intervention strategies.

The Q-matrix serves as a framework linking each SCWT item type to the cognitive attributes required for accurate responses.

(a) Congruent trials may require only basic word recognition and color identification.
(b) Incongruent trials require both color identification and inhibitory control.
(c) Neutral trials may emphasize processing speed with minimal inhibitory demand.

In the NPCD model (34), because no established normative criteria currently exist for Stroop-based cognitive mastery following acute exercise interventions, the present study adopted a relative mastery framework rather than an absolute mastery framework. Relative mastery was first determined according to group performance benchmarks within each exercise condition. Participants performing at or above the group mean were classified as demonstrating relative mastery for the corresponding attribute, whereas those below the group mean were classified as relative non-mastery. Subsequently, attribute states were dichotomized within the NPCD framework, where relative mastery was coded as 1 and relative non-mastery was coded as 0. This criterion was adopted in the present study to facilitate relative cognitive classification in the absence of established criterion-referenced standards. Participant demographic and anthropometric characteristics are presented in Table 2.

**Table 2:** Participant demographic and anthropometric characteristic, mean (S.D.).

Characteristics	Participants Mean (S.D.)
Age (years)	18.72 (0.36)
Height (cm)	170.58 (5.63)
Weight (kg)	71.82 (8.76)
Body Mass Index (kg/m^2^)	24.69 (2.37)
Muscle mass (kg)	34.73 (5.08)
Body fat mass (kg)	15.46 (5.71)
Body fat percentage (%)	18.60 (5.36)
VO_2peak_ (mL kg^−1^ min^−1^)	46.25 (6.18)

### Procedures

Participants were assigned to four groups and completed three SCWT task types-static, mobile, and complex, via a mobile device (Figure 3).

**Figure 3:**
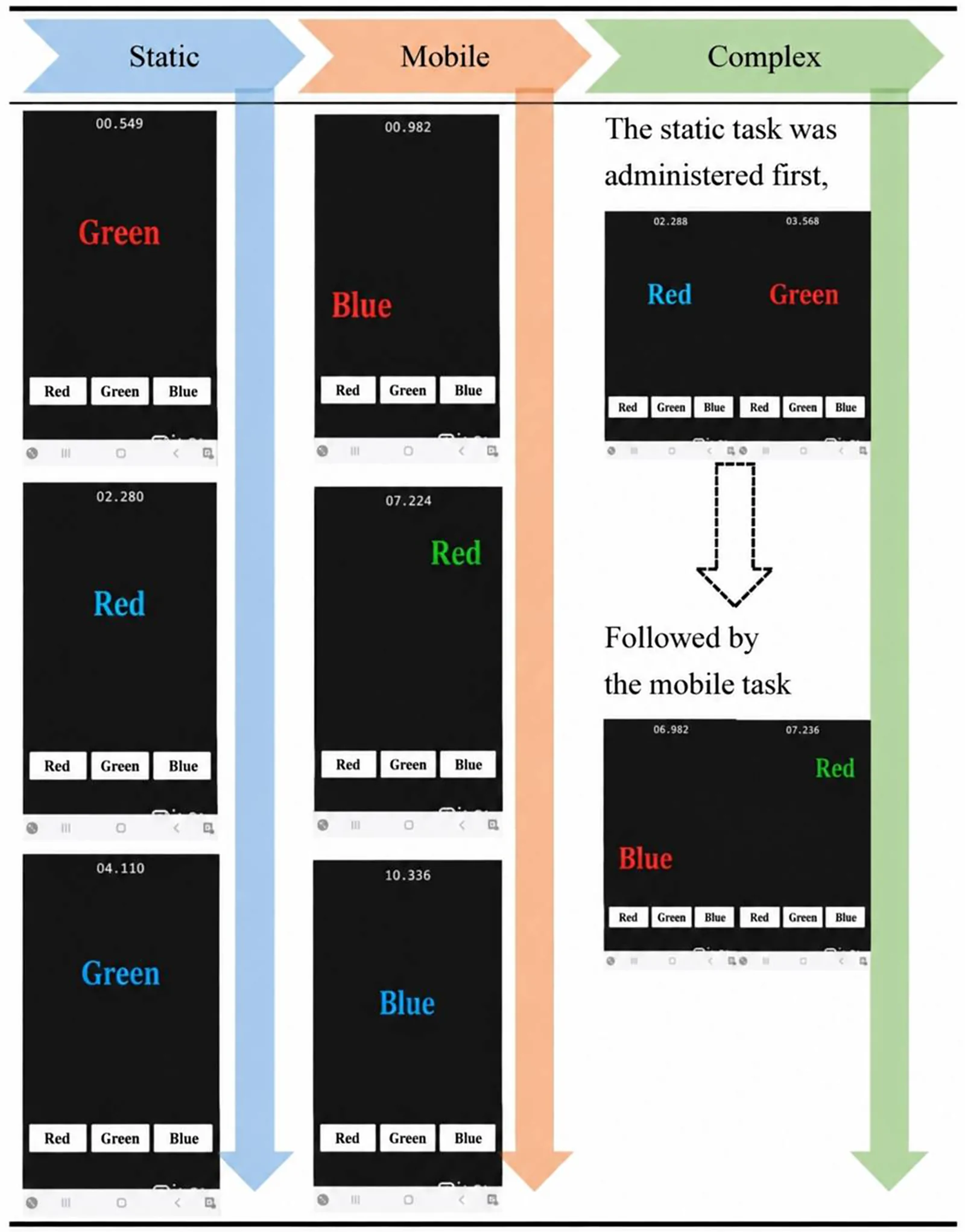
Procedure for the three types of SCWT tasks.

Group means were used to define relative mastery: participants scoring at or above the mean on the SCWT were classified as relative mastery, and those below as non-relative mastery. The NPCD validated the Q-matrix and enabled fine-grained, assumption-free analysis of cognitive attributes. Non-relative mastery participants received targeted remedial instruction, aligning the intervention with individualized cognitive diagnostic standards. Performance was based on each group's average completion time, with shorter times indicating better outcomes, so identical group means were unnecessary despite potential challenges for inter-group comparisons

### Statistical analysis

Descriptive statistics summarized participant and exercise data. Statistical analyses were conducted using SPSS 25. Descriptive statistics were calculated for all variables. Differences among the four groups were analyzed using one-way analysis of covariance (ANCOVA), with baseline scores entered as the covariate. When a significant main effect was observed, Bonferroni-adjusted *post hoc* comparisons were performed. Homogeneity of variance was assessed using Levene's test. For each SCWT condition, an ANCOVA was conducted with four group as the independent variable, post-test performance as the dependent variable, and pre-test scores entered as covariates. Significant effects were followed by Bonferroni-adjusted *post hoc* comparisons. Effect sizes were reported as partial eta squared (*η*^2^), and statistical significance was set at *α* = .05, with pre-test scores as covariates and post-test performance as the dependent variable. Although a mixed ANOVA can simultaneously evaluate Group × Time interactions, the primary objective of this study was to compare post-test performance among exercise groups while controlling for baseline differences. Therefore, one-way ANCOVA with baseline scores as covariates was considered the most appropriate analytical approach.

## Results

Completion time was the primary performance indicator, with shorter durations indicating better outcomes. Significant group differences were observed. Exercise intensity data, including heart rate, %HRmax, and RPE, are presented in Table 3. Descriptive statistics for pre- and post-test SCWT performance, including means and standard deviations, are presented in Table 4. Bonferroni-adjusted pairwise comparisons, including adjusted mean differences, standard errors, *p*-values, and 95% confidence intervals, are presented in Table 5, forming the basis for further analysis. Accuracy was therefore not analyzed as an independent outcome variable because incorrect responses did not contribute separately to task completion.

**Table 3:** Exercise intensity during intervention.

Group	RPE (Mean ± SD)	Heart Rate (Mean ± SD, bpm)	%HRmax (Mean ± SD)
RE	11.2 ± 0.8	99.3 ± 2.7	50.4 ± 1.6
AE	11.6 ± 0.7	120.2 ± 2.5	60.2 ± 1.8
GE	11.8 ± 0.6	141.4 ± 2.9	70.3 ± 1.9
NE	N/A	N/A	N/A

**Table 4:** Descriptive statistics for SCWT performance across conditions.

Task	Group	Pre-test Mean (SD)	Post-test Mean (SD)
Static	GE	54.93 (0.70)	51.02 (0.52)
Static	RE	54.91 (0.67)	52.87 (0.68)
Static	AE	54.91 (0.67)	52.40 (0.56)
Static	NE	57.23 (0.50)	55.99 (0.63)
Mobile	GE	56.10 (0.69)	53.21 (0.75)
Mobile	RE	57.43 (0.68)	54.97 (0.95)
Mobile	AE	56.41 (0.67)	54.78 (0.92)
Mobile	NE	58.03 (0.50)	57.14 (0.63)
Complex	GE	57.41 (0.67)	56.39 (0.72)
Complex	RE	58.93 (0.68)	57.41 (0.55)
Complex	AE	58.10 (0.69)	56.08 (0.74)
Complex	NE	58.53 (0.50)	57.86 (0.44)

**Table 5:** Bonferroni-adjusted pairwise comparisons across attention tasks.

Task	Comparison	Adjusted Mean Difference	SE	*p*-value	95% CI
Static	AE vs. GE	1.400	0.156	<.001	[0.974, 1.827]
	AE vs. NE	−1.995	0.254	<.001	[−2.691, −1.299]
	AE vs. RE	−0.469	0.156	.024	[−0.895, −0.043]
	GE vs. NE	−3.395	0.253	<.001	[−4.087, −2.703]
	GE vs. RE	−1.869	0.156	<.001	[−2.295, −1.443]
	NE vs. RE	1.526	0.254	<.001	[0.830, 2.222]
Mobile	AE vs. GE	1.265	0.210	<.001	[0.690, 1.840]
	AE vs. NE	−0.799	0.277	.034	[−1.558, −0.040]
	AE vs. RE	0.793	0.238	.009	[0.143, 1.443]
	GE vs. NE	−2.064	0.303	<.001	[−2.893, −1.235]
	GE vs. RE	−0.472	0.257	.431	[−1.177, 0.232]
	NE vs. RE	1.592	0.218	<.001	[0.996, 2.188]
Complex	AE vs. GE	−0.883	0.132	<.001	[−1.244, −0.522]
	AE vs. NE	−1.409	0.127	<.001	[−1.757, −1.062]
	AE vs. RE	−0.621	0.136	<.001	[−0.993, −0.248]
	GE vs. NE	−0.526	0.145	.004	[−0.924, −0.129]
	GE vs. RE	0.262	0.161	.659	[−0.179, 0.704]
	NE vs. RE	0.789	0.127	<.001	[0.442, 1.135]

Pairwise comparisons were adjusted using the Bonferroni correction. Mean differences are based on estimated marginal means after controlling for pretest scores.

### Exercise intensity

Heart rate monitoring confirmed that participants exercised within the prescribed intensity ranges. The mean heart rates were 99.3 ± 2.7 bpm in the RE group, 120.2 ± 2.5 bpm in the AE group, and 141.4 ± 2.9 bpm in the GE group, corresponding to approximately 50.4 ± 1.6%, 60.2 ± 1.8%, and 70.3 ± 1.9% of HRmax, respectively. The mean ratings of perceived exertion (RPE) ranged from 10 to 13 across all exercise groups, indicating that the prescribed exercise intensities were successfully achieved.

### Static attention

After adjusting for baseline (pre-test) performance, ANCOVA revealed significant group effects on static attention [*F*(3,55) = 84.389, *p* < .001, *η^2^* = .822]. Levene's test confirmed homogeneity of variance [*F*(3,56) = 0.610, *p* = .611]. Adjusted means ranked GE (*M* = 51.41) < AE (*M* = 52.81) < RE (*M* = 53.28) < NE (*M* = 54.80), with GE outperforming all others, indicating superior static attention. Bonferroni-adjusted pairwise comparisons revealed significant differences among all groups (Table 4). GE demonstrated significantly faster reaction times than AE, RE, and NE.

### Mobile attention

Levene's test confirmed homogeneity [*F*(3,56) = 1.284, *p* = .289]. ANCOVA showed a significant group effect on performance [*F*(3,55) = 28.466, *p* < .001, *η^2^*=.608] after controlling for pretest scores. Adjusted means ranked GE (*M* = 54.08) < RE (*M* = 54.55) < AE (*M* = 55.34) < NE (*M* = 56.14). *Post hoc* tests showed GE outperformed AE and NE, while NE had the longest reaction times. Bonferroni comparisons indicated that GE outperformed AE and NE, whereas no significant difference was observed between GE and RE (*p* = .431).

### Complex attention

Levene's test confirmed homogeneity [*F*(3,56) = 1.859, *p* = .147]. ANCOVA revealed significant group effects after controlling for pretest scores [*F*(3,55) = 44.886, *p* < .001, *η*^2^=.710]. Adjusted means ranked AE (*M* = 56.21) < RE (*M* = 56.83) < GE (*M* = 57.09) < NE (*M* = 57.62). AE outperformed all other groups, while NE showed the poorest performance. Bonferroni-adjusted comparisons showed that AE achieved significantly better performance than GE, RE, and NE. No significant difference was observed between GE and RE (*p* = .659). Visual summaries include a bar chart (Figure 4), line chart (Figure 5). Bonferroni-adjusted pairwise comparisons for all three attention tasks are presented in Table 4. For the static attention task, all pairwise comparisons reached statistical significance. For the mobile and complex attention tasks, no significant differences were observed between the agility and resistance exercise groups, whereas all remaining comparisons were statistically significant after Bonferroni adjustment.

**Figure 4:**
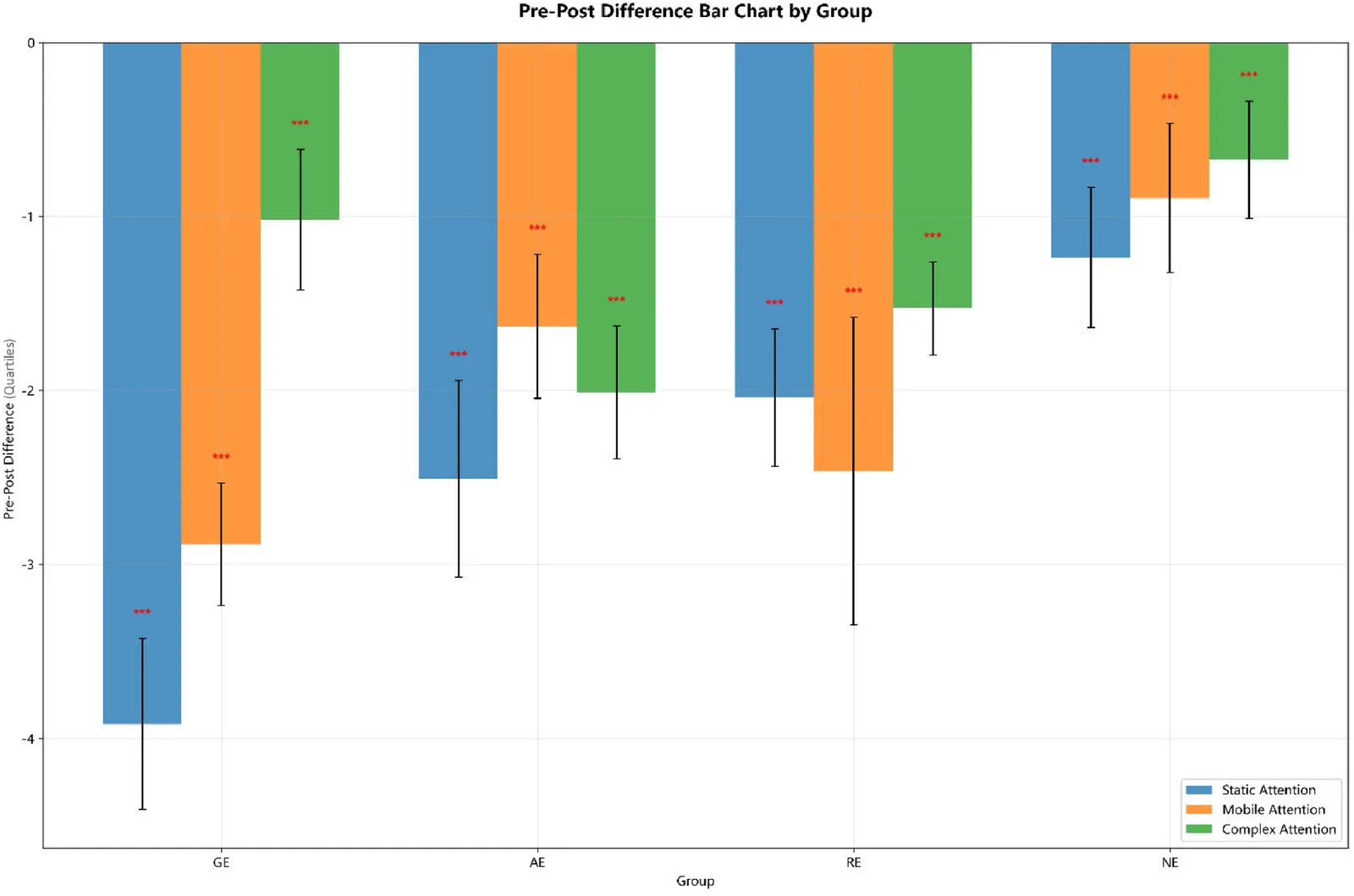
Shows a bar chart comparing pre- and post-test differences.

**Figure 5:**
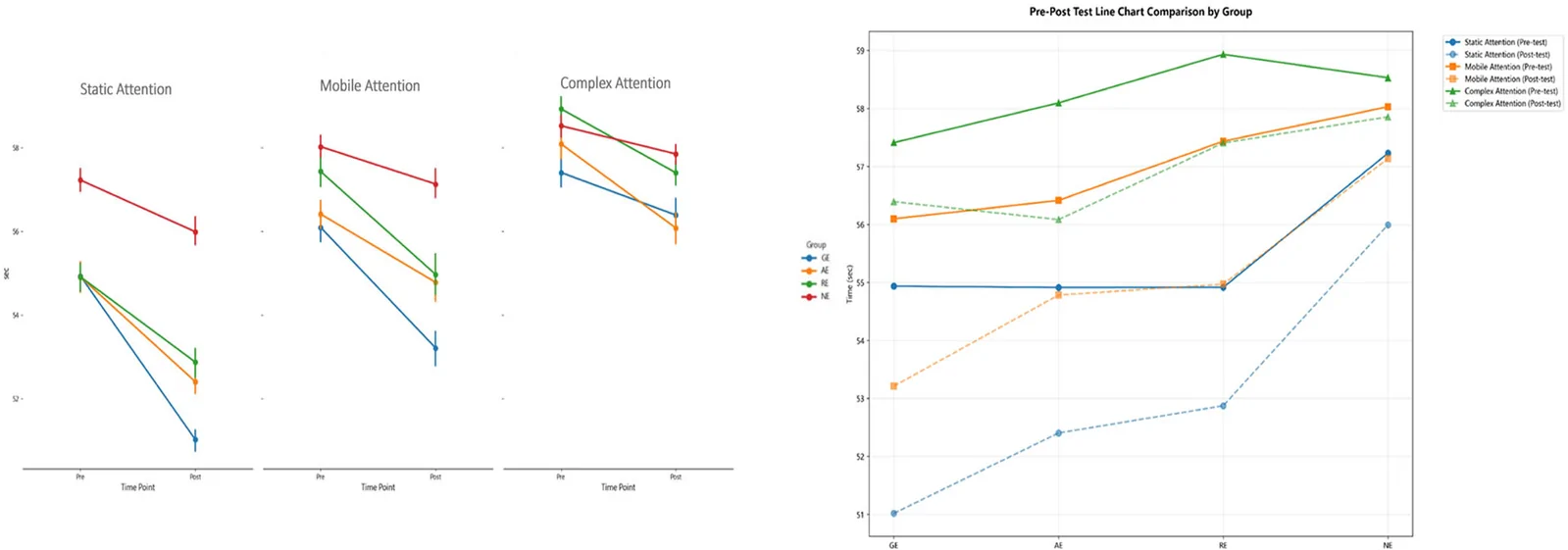
Illustrates a line chart depicting pre- and post-test trends.

Overall, agility training (GE) produced the greatest gains in static and mobile attention, whereas aerobic exercise (AE) most enhanced complex attention. These results suggest agility training improves attentional control, while aerobic exercise supports integrative cognitive processing (35, 36). Following the procedures, NPCD analysis was performed. The mean completion time of each group served as the performance benchmark, with relative mastery coded as “1” and non- relative mastery as “0.” Q-matrix analysis was then applied to identify potentially non-mastered attributes for exploratory hypothesis generation and future individualized intervention planning, which were aligned with the original exercise tasks. This method aligns with current applications of the NPCD models in educational and cognitive assessment research (32, 37). Applying an NPCD with a validated Q-matrix enables fine-grained diagnosis of cognitive attributes affected by different exercise interventions, providing skill-level insights beyond overall performance measures, As shown in Table 6, the data provide valuable information.

**Table 6:** Q-Matrix results of this study.

Q-Matrix(GE)					Q-Matrix(RE)					Q-Matrix(AE)					Q-Matrix(NE)				
Time to Criterion (seconds)					Time to Criterion (seconds)					Time to Criterion (seconds)					Time to Criterion (seconds)				
	Static	Mobile	Complex			Static	Mobile	Complex			Static	Mobile	Complex			Static	Mobile	Complex	
	51.02(s)	53.21(s)	56.39(s)			52.87(s)	54.97(s)	57.41(s)			52.40(s)	54.78(s)	56.08(s)			55.99(s)	57.14(s)	57.86(s)	
Group	Static	Mobile	Com-	Total	Group	Static	Mobile	Com	Total	Group	Static	Mobile	Com-	Total	Group	Static	Mobile	Com-	Total
GE1	0	0	0	0	RE1	1	0	0	1	AE1	0	1	0	1	NE1	0	1	0	1
GE2	0	1	1	2	RE2	0	0	0	0	AE2	0	0	1	1	NE2	1	1	1	3
GE3	0	1	1	2	RE3	0	0	1	1	AE3	1	0	1	2	NE3	0	1	0	1
GE4	0	0	1	1	RE4	1	0	0	1	AE4	0	0	0	0	NE4	1	1	0	2
GE5	1	0	0	1	RE5	0	1	1	2	AE5	1	1	0	2	NE5	0	1	0	1
GE6	0	1	1	2	RE6	1	1	0	2	AE6	0	1	1	2	NE6	1	1	1	3
GE7	0	1	0	1	RE7	0	0	1	1	AE7	1	0	0	1	NE7	1	0	1	2
GE8	1	1	1	3	RE8	1	1	1	3	AE8	0	1	0	1	NE8	1	1	1	3
GE9	1	1	1	3	RE9	1	0	0	1	AE9	0	0	1	1	NE9	0	1	0	1
GE10	0	1	1	2	RE10	0	1	1	2	AE10	0	1	1	2	NE10	1	1	0	2
GE11	0	1	1	2	RE11	1	1	0	2	AE11	0	0	1	1	NE11	0	0	0	0
GE12	1	0	0	1	RE12	0	1	1	2	AE12	1	0	0	1	NE12	1	1	1	3
GE13	0	1	1	2	RE13	1	0	0	1	AE13	1	1	0	2	NE13	1	0	0	1
GE14	1	0	1	2	RE14	0	1	1	2	AE14	1	0	1	2	NE14	0	0	0	0
GE15	0	0	1	1	RE15	1	0	0	1	AE15	1	1	0	2	NE15	0	1	0	1

The results indicated that both static and mobile attention were most strongly associated with the agility exercise group, while the complex state was strongly linked to AE ability. Q-matrix analysis was then applied to exploratorily classify relative cognitive strengths and weaknesses, which may provide preliminary guidance for future individualized intervention planning. For example, participant RE5 (“0 1 1”) may potentially benefit from agility-related training; however, this interpretation should be regarded as exploratory and hypothesis-generating only. Conversely, participant AE13 (“1 1 0”) showed a relative non-mastery profile in the complex state, suggesting that additional aerobic training may be considered in future intervention planning. This coding procedure was applied uniformly across participants. The cognitive diagnostic results provided preliminary, exploratory information regarding participants who may potentially benefit from future individualized intervention planning. However, the specific content, implementation, and effectiveness of such remedial interventions represent a separate research topic and were not examined within the scope of the present study.

## Discussion

The modality-specific effects observed in the present study may be explained by differences in the physiological and cognitive demands imposed by each exercise type. Agility exercise requires rapid processing of environmental information, frequent attentional shifts, motor planning, and inhibitory control. Consequently, agility training may preferentially activate frontoparietal networks associated with selective attention and executive control, resulting in enhanced performance during static and mobile Stroop conditions. In contrast, aerobic exercise has been associated with increased cerebral blood flow, elevated arousal, and transient increases in neurotrophic factors such as BDNF, which may facilitate broader cognitive integration and explain the superior performance observed during complex attention tasks. Resistance exercise may contribute through neuromuscular and endocrine responses; however, its cognitive demands are generally lower than those of agility exercise. Acute Exercise Mechanism may influence cognitive performance through several complementary mechanisms. Previous studies have suggested that a single bout of exercise can increase cortical arousal, enhance cerebral oxygenation, and improve neural efficiency within prefrontal regions responsible for executive function. Exercise-induced elevations in catecholamines and neurotrophic factors may further facilitate attentional processing and response selection. The present findings support the view that exercise modalities incorporating greater perceptual–motor complexity may generate larger short-term cognitive benefits than exercise modes relying primarily on metabolic demand alone.

Although the present study showed large effects of exercise modality on Stroop performance, the magnitude of the observed effect sizes should be interpreted with caution. The partial eta squared values obtained in the ANCOVA models were high (*η*^2^ = .608–.822), exceeding the small-to-moderate effects commonly reported in previous acute exercise studies. Meta-analytic evidence has suggested that the overall effect of acute exercise on cognitive performance is generally small, although larger effects may occur depending on exercise intensity, cognitive task demands, and timing of post-exercise assessment. Therefore, the inflated effect sizes in the present study may be partly attributable to the homogeneous male adolescent sample, small group size, restricted within-group variability, One possible explanation for the observed improvements in Stroop performance is the presence of learning effects theory, whereby performance improves because of repeated exposure to the testing procedure rather than the intervention itself (38). Repeated administration of cognitive tests may increase familiarity with task requirements and response strategies, thereby contributing to improved post-test performance. These findings should therefore be considered preliminary and require replication in larger, more diverse samples using preregistered protocols and confidence intervals for effect-size estimates. Overall, the present findings support previous evidence indicating that different acute exercise modalities exert distinct cognitive effects. These modality-specific patterns are consistent with previous studies suggesting that agility exercise preferentially enhances attentional flexibility and inhibitory control, whereas aerobic exercise facilitates broader cognitive integration through increased cerebral blood flow and exercise-induced neurotrophic responses (23, 35, 39, 40). BDNF enhances long-term potentiation by improving synaptic efficiency and promoting dendritic growth. Both acute and chronic exercise elevate circulating BDNF levels, thereby enhancing executive function, attentional control, and emotional regulation (41, 42). Although less pronounced, resistance training yielded moderate benefits, consistent with evidence that neuromuscular and endocrine adaptations enhance working memory and inhibitory control. Besides, Although accuracy was initially included as an outcome measure, accuracy scores were uniformly high across all groups and conditions, indicating a potential ceiling effect. Because participants were required to achieve adequate task comprehension before formal testing and could only proceed after providing correct responses, variability in accuracy was substantially restricted. Consequently, reaction time was considered the more sensitive indicator of exercise-related cognitive changes. Nevertheless, future studies should employ more challenging cognitive paradigms to avoid ceiling effects and allow simultaneous evaluation of both speed and accuracy. It should be noted that the mastery classifications reported in this study represent relative mastery rather than absolute mastery. The present study employed a Relative Mastery Approach because validated normative benchmarks and criterion-referenced standards for Stroop-based cognitive diagnostic assessment following acute exercise interventions are currently lacking. Consequently, participants’ cognitive attribute mastery was evaluated relative to group-specific performance benchmarks rather than against absolute mastery criteria. Therefore, the NPCD results should be interpreted as exploratory classifications intended to support individualized remedial instruction rather than definitive cognitive diagnoses. Accordingly, the present mastery classifications should not be interpreted as evidence of absolute cognitive proficiency or deficiency, but rather as relative comparisons within the current sample.

### Physiological mechanisms

The distinct effects of exercise modalities reflect their physiological mechanisms. Agility training, emphasizing neuromuscular coordination, proprioception, and perceptual decision-making, engages frontoparietal networks underlying attentional flexibility (43, 44). Aerobic exercise enhances cardiovascular efficiency and oxygen delivery, promoting cortical oxygenation and neurogenesis in hippocampal and prefrontal regions (45). Resistance training elicits endocrine responses and structural adaptations that modestly support executive function, though less effectively than agility or aerobic exercise.

### Practical implications

For individuals with limited exercise habits, structured physical activity can substantially improve cognition. Agility training enhances rapid inhibitory control relevant to learning and sports, whereas aerobic exercise benefits sustained attention and information processing. Resistance training offers complementary gains when combined in multimodal programs.

### Limitations and future directions

Several limitations should be noted. The homogeneous male adolescent sample limits generalizability across ages, genders, and cultures. Only acute exercise effects were examined, leaving long-term adaptations unaddressed. As the Stroop task assesses a limited aspect of executive function, future studies should use broader cognitive batteries. Longitudinal and neuroimaging research is needed to clarify causal mechanisms and potential synergistic effects of combined exercise modalities. The present NPCD classifications are intended primarily for hypothesis generation and should be validated using externally referenced mastery criteria in future studies. In addition, although the mobile SCWT application has demonstrated acceptable validity in previous studies, validation evidence specifically involving adolescent athletic populations remains limited. Therefore, the applicability of the present findings to this population should be interpreted with appropriate caution, and further validation studies are warranted.

## Conclusion

The findings highlight the modality-specific nature of exercise-induced cognitive benefits. Agility training effectively enhances attentional flexibility, aerobic exercise optimizes sustained and integrative attention, and resistance training provides supplementary effects. A unique contribution of the present study lies in its interdisciplinary integration of exercise science and cognitive diagnostic assessment. Beyond investigating the cognitive effects of different exercise modalities, the study employed the NPCD and Q-matrix analysis to exploratorily classify relative cognitive attributes. These classifications should be interpreted as exploratory and hypothesis-generating rather than as externally validated cognitive diagnoses. This interdisciplinary approach enabled the identification of potentially non-mastered attributes and facilitated the development of individualized remedial instructional strategies. Future interventions should tailor exercise prescriptions to targeted cognitive goals and promote activity among sedentary populations. Furthermore, studies using larger samples and externally referenced mastery criteria may provide clearer insights into the cognitive effects of exercise modalities and sport-specific motor–cognitive training strategies.

## Data Availability

The original contributions presented in the study are included in the article/Supplementary Material, further inquiries can be directed to the corresponding author/s.
